# Asynchronous Code Division Multiplexing-Based Visible Light Positioning and Communication Network Using Successive Interference Cancellation Decoding

**DOI:** 10.3390/s24175609

**Published:** 2024-08-29

**Authors:** Zhongxu Liu, Xiaodi You, Changyuan Yu

**Affiliations:** 1 Department of Electrical and Electronic Engineering, The Hong Kong Polytechnic University, Kowloon, Hong Kong 999077, China; zhongxu.liu@connect.polyu.hk (Z.L.); changyuan.yu@polyu.edu.hk (C.Y.); 2 School of Electronic and Information Engineering, Soochow University, Suzhou 215000, China

**Keywords:** visible light positioning (VLP), visible light positioning and communication (VLPC), successive interference cancellation decoding (SICD), asynchronous code division multiplexing (ACDM)

## Abstract

In the evolving landscape of sixth-generation wireless communication, the integration of visible light communication (VLC) and visible light positioning (VLP), known as visible light positioning and communication (VLPC), emerges as a pivotal technology. This study addresses the challenges of asynchronous code division multiplexing (ACDM) in VLPC networks, focusing on the enhancement of data transmission quality and positioning accuracy. Firstly, we propose an orthogonal pseudo-random code (OPRC) for ACDM-based VLP systems. Leveraging its excellent correlation properties, VLP signals preserve orthogonality even amidst asynchronous transmissions, achieving sub-centimeter average positioning errors. Next, by combining OPRC with successive interference cancellation decoding (SICD), we propose an enhanced ACDM-based VLPC system that utilizes OPRC for improved signal orthogonality and SICD for progressive elimination of multiple access interference (MAI) among VLPC signals. The results show substantial improvements in bit-error rate (BER) and positioning error (PE), approaching the performance levels observed in synchronized VLPC systems. Specifically, the SICD-OPRC scheme reduces average BER to 4.3 × 10^−4^ and average PE to 2.7 cm, demonstrating its robustness and superiority in complex asynchronous scenarios.

## 1. Introduction

With the unprecedented popularization of intelligent devices and the rapid development of Internet of Things (IoT) technologies, future wireless communication networks are expected to offer large communication capacity, ultra-low latency communication, large-scale autonomous connectivity, and centimeter-level positioning in the sixth-generation (6G) era [1,2]. Motivated by the widespread deployment of power-efficient light-emitting diodes (LEDs), visible light communication (VLC) has become a promising technology for 6G communication due to its advantages such as unregulated tremendous spectrum resources, energy conservation, high data confidentiality, and immunity to electromagnetic interference [3,4]. Meanwhile, visible light positioning (VLP) is another functionality brought by LEDs, offering high positioning accuracy up to the centimeter level [5,6]. As is foreseen, future LED-based lighting facilities can be a part of 6G infrastructure to provide multi-services, including illumination, communication, and positioning.

Achieving simultaneous high-speed VLC and high-accurate VLP is essential for enhancing the capabilities of IoT applications, such as logistics and service robots, robotic arms, and virtual reality devices [7,8]. To address this requirement, extensive studies have been conducted to integrate VLC and VLP, and the concept of visible light positioning and communication (VLPC) was proposed. In VLPC systems, enabling the receiver to differentiate between VLC and VLP signals necessitates the adoption of multiplexing techniques. Two widely used methods for achieving this are time division multiplexing (TDM) and frequency division multiplexing (FDM). In TDM-based VLPC systems, the total time resource is divided into multiple time slots, allocating some for VLC and others (typically at least three for trilateration) for VLP [9,10,11]. However, this approach introduces latency for both VLC and VLP services and diminishes the efficiency of VLC transmission. Alternatively, in FDM-based VLPC systems, the available frequency spectrum is split into dedicated VLC and VLP sub-bands [12,13,14]. However, since the bandwidth of commercial LEDs is usually a few tens of MHz, VLP will limit the capacity of VLC, and additional guard bands for separating VLPC signals will reduce the spectral utilization. In addition, FDM-based solutions suffer from high out-of-band interference and peak-to-average power ratio, degrading the system performance [15]. Given these limitations, a hybrid heterogeneous signal extraction scheme was introduced to the VLPC system, where a low-pass complementary metal-oxide-semiconductor (CMOS) image sensor and a high-bandwidth photodetector (PD) are used to capture the low-speed VLP signals and high-speed VLC signals, respectively [16]. However, this will undoubtedly increase the hardware cost and system complexity. In [17], a new VLPC system is proposed, which uses the average energy of VLC signals combined with an artificial neural network (ANN) to predict the user’s mobility path. However, this system requires the knowledge of the user’s initial position, which limits its practicality. The idea of using average energy of VLC signals for positioning is also used in a VLPC system based on a solar cell array receiver [18], but the complex receiver array increases hardware costs, and the low bandwidth of solar cells restricts the communication rate.

Recently, different approaches based on code division multiplexing (CDM) have been employed in VLC [19,20,21], VLP [22,23,24], and VLPC [25,26] systems. By utilizing the orthogonality characteristic of various orthogonal codewords, CDM-based approaches are able to distinguish different signals at the receiver and thus can transmit multiple VLC and VLP signals in the same time slot and frequency band efficiently. However, all these approaches are based on the assumption of synchronous CDM (i.e., no time delays among transceivers) [19,20,21,22,23,24,25] or quasi-synchronous CDM (i.e., very slight time delays exist among transceivers) [26]. Ideally, for synchronized CDM systems, the cross-correlation of the orthogonal codewords is zero [27], which guarantees the perfect orthogonality between VLC and VLP signals. However, in practice, LED transmitters are typically controlled independently, leading to asynchronous VLC and VLP signal emissions. Moreover, varying distances between LED transmitters and the receiver result in differing signal propagation times. Therefore, when the CDM-based VLC and VLP signals emitted from different LED transmitters arrive at the receiver, time delays always exist among CDM transceivers. These time delays are non-negligible as compared with the chip length of a CDM codeword, leading to the scenario of asynchronous CDM (ACDM). For ACDM signals, the cross-correlation of the orthogonal codewords increases due to the chip shift, which deteriorates the orthogonality between VLC and VLP signals. This causes severe multiple access interference (MAI) during signal decoding, adversely impacting VLC and VLP performance [28,29]. Although different schemes were proposed to alleviate the MAI of ACDM signals in radio frequency communication (RFC) systems [30,31,32], these schemes are mainly designed for multi-user communication and cannot support high-accurate positioning. Furthermore, given the distinct nature of the VLC channel compared to RFC channels, RFC-designed schemes are not directly applicable to VLPC systems. Therefore, few efforts have been made to improve the performance and robustness of ACDM-based VLPC systems. Motivated by this gap, our work focuses on enhancing the VLPC scheme to mitigate ACDM-induced MAI, aiming to handle significant time delays among CDM transceivers.

In this paper, we present a novel solution for enhancing the performance of the ACDM-based VLPC network. Firstly, we propose a new type of orthogonal codeword called orthogonal pseudo-random code (OPRC), designed for the ACDM-based VLP system. Building on our previous conceptualization of the OPRC-VLP scheme [33], we conduct a detailed investigation into the OPRC-VLP system based on ACDM through simulations and experiments. Next, we enhance the OPRC-VLP scheme to include VLC capabilities, resulting in the OPRC-VLPC scheme. This scheme operates within a multi-cell VLPC network, where LEDs within the same cell are managed by a unified controller to maintain synchronization, while LEDs across different cells are controlled independently, leading to asynchronous transmission. In such asynchronous settings, since the coding contains user information, differences between adjacent data bits can exacerbate MAI. Thus, a successive interference cancellation decoding (SICD) technique is introduced to reduce the influence of MAI and improve the system performance. Simulation results confirm the effectiveness of the SICD-OPRC scheme, demonstrating its capability to maintain high-quality data transmission and precise positioning in asynchronous VLPC networks. The main contributions of this paper are summarized as follows:

(1) We present the mathematical formulation for generating the OPRC and analyze its correlation functions, demonstrating that it exhibits excellent correlation properties. Specifically, its cross-correlation is always zero at any chip shift, which preserves the orthogonality during asynchronous transmissions. Results indicate that the superior correlation properties of OPRC offer greater resilience to MAI compared to OZCZ codes as proposed in [26].
(2) Comprehensive simulations and experiments are conducted for the OPRC-VLP scheme in ACDM-based systems. The utilization of OPRC enables clear differentiation of VLP signals from individual LEDs at the receiver, free from MAI. Thus, the OPRC-VLP scheme achieves sub-centimeter precision positioning without synchronization between transmitters, both in simulation and experiment.
(3) We further propose an ACDM-based VLPC scheme that simultaneously offers VLP and VLC capabilities. To mitigate the impact of MAI on decoding, we introduce an SICD technique that progressively eliminates interfering signals, optimizes decoding accuracy, and enhances VLC and VLP performance.
(4) Extensive simulations are conducted to compare the performance of VLPC systems based on SICD-OPRC, OPRC, and OZCZ. Specifically, in asynchronous transmission scenarios, the OZCZ-based scheme achieves an average bit-error rate (BER) of 4.0 × 10^−2^ and an average positioning error (PE) of 32.5 cm. The OPRC-based scheme improves these metrics to an average BER of 2.3 × 10^−2^ and an average PE of 19.9 cm. Most notably, the SICD-OPRC scheme further reduces the average BER to 4.3 × 10^−4^ and the average PE to 2.7 cm, nearly matching the BER and PE levels observed in synchronized VLPC systems.

The rest of this paper is organized as follows. Section 2 introduces the generation process of OPRC and provides a detailed analysis of its correlation characteristics. Section 3 focuses on the operation mechanism of the OPRC-VLP system, detailing the simulation and experimental setup and offering a comprehensive interpretation of the collected data to validate the system’s performance. Section 4 introduces the VLPC network, emphasizing the framework of the SICD-OPRC-VLPC system, the construction of its simulation model, and simulations in both synchronous and asynchronous environments. Finally, Section 5 concludes the paper.

## 2. Construction and Correlation Properties of OPRC

To create codewords that are better suited for ACDM-based systems, we integrate cyclic orthogonal Walsh–Hadamard codes (COWHCs) with m-sequences. One significant characteristic of COWHCs is that their cross-correlation values remain zero under any chip shift, effectively reducing interference between different codes. Meanwhile, m-sequences exhibit remarkable advantages in auto-correlation properties, featuring a prominent main peak and low side lobes, which facilitate precise signal identification and synchronization. Our objective is to combine the excellent cross-correlation performance of COWHCs with the superior auto-correlation properties of m-sequences to design a novel codeword. This new type of code will have enhanced correlation characteristics, providing improved signal distinguishability and interference resistance in asynchronous transmission systems. Now, we describe the process of constructing OPRC codes.

First, we define ***h****_i_* = [*h_i_*_1_, …, *h_iL_h__*] as a COWHC sequence of length *L_h_*, and arrange *N* different sequences ***h****_i_* (*i* = 1, …, *N*) to form an *N* × *L_h_* matrix, which can be represented as:

(1) $$A=\left[\begin{array}{c} A_{1}\\ \vdots \\ A_{N} \end{array}\right]=\left[\begin{array}{c} h_{1}\\ \vdots \\ h_{N} \end{array}\right]=\left[\begin{array}{ccc} h_{11} & \cdots & h_{1L_{h}}\\ \vdots & \vdots & \vdots \\ h_{N} & \cdots & h_{NL_{h}} \end{array}\right]$$

Then, we define ***m*** = [*m*_1_, …, *m_Lm_*] as an *m*-sequence of length *L_m_*, and arrange *N* same sequence ***m*** to form an *N* × *L_m_* matrix, which can be represented as:

(2) $$B=\left[\begin{array}{c} B_{1}\\ \vdots \\ B_{N} \end{array}\right]=\left[\begin{array}{c} m\\ \vdots \\ m \end{array}\right]=\left[\begin{array}{ccc} m_{1} & \cdots & m_{L_{m}}\\ \vdots & \vdots & \vdots \\ m_{1} & \cdots & m_{L_{m}} \end{array}\right]$$

Next, we stack matrix ***A*** continuously along the column direction *L_m_* times to obtain matrix ***H*** and stack matrix ***B*** continuously along the column direction *L_h_* times to obtain matrix ***M***. Thus, these two matrices have the same dimensions *N* × *L_h_L_m_*, which is written by:

(3) $$\left\{\begin{array}{c} H=\left[\begin{array}{ccc} A & \cdots & A \end{array}\right]=\left[\begin{array}{lllll} H_{11} & \cdots & H_{1L_{h}} & \cdots & H_{1\left(L_{m}\times L_{h}\right)}\\ \vdots & \vdots & \vdots & \vdots & \vdots \\ H_{N1} & \cdots & H_{NL_{h}} & \cdots & H_{N\left(L_{m}\times L_{h}\right)} \end{array}\right]=\left[\begin{array}{lllll} h_{11} & \cdots & h_{1L_{h}} & \cdots & h_{1L_{h}}\\ \vdots & \vdots & \vdots & \vdots & \vdots \\ h_{N1} & \cdots & h_{NL_{h}} & \cdots & h_{NL_{h}} \end{array}\right]\\ M=\left[\begin{array}{ccc} B & \cdots & B \end{array}\right]=\left[\begin{array}{lllll} M_{11} & \cdots & M_{1L_{m}} & \cdots & M_{1\left(L_{h}\times L_{m}\right)}\\ \vdots & \vdots & \vdots & \vdots & \vdots \\ M_{N1} & \cdots & M_{NL_{m}} & \cdots & M_{N\left(L_{h}\times L_{m}\right)} \end{array}\right]=\left[\begin{array}{lllll} m_{1} & \cdots & m_{L_{m}} & \cdots & m_{L_{m}}\\ \vdots & \vdots & \vdots & \vdots & \vdots \\ m_{1} & \cdots & m_{L_{m}} & \cdots & m_{L_{m}} \end{array}\right] \end{array}\right.$$

Finally, we generate a new matrix ***S*** by computing the Hadamard product of matrices ***H*** and ***M***. In this process, each element of ***S*** is obtained by multiplying the corresponding elements of ***H*** and ***M*** at the same positions. Therefore, ***S*** can be represented as follows:

(4) $$S=H⊙M=\left[\begin{array}{c} S_{1}\\ \vdots \\ S_{N} \end{array}\right]=\left[\begin{array}{ccc} H_{11}M_{11} & \cdots & H_{1\left(L_{m}\times L_{h}\right)}M_{1\left(L_{h}\times L_{m}\right)}\\ \vdots & \vdots & \vdots \\ H_{N1}M_{N1} & \cdots & H_{N\left(L_{m}\times L_{h}\right)}M_{N\left(L_{h}\times L_{m}\right)} \end{array}\right],$$

where ***S****_i_* represents a specific codeword from the generated set of OPRC with a length of *Ls* = *L_h_* × *L_m_*.

According to [34], when two code sequences are multiplied, the correlation function of the resulting sequence is equal to the product of the individual correlation functions of the two original code sequences. Thus, the correlation function of the OPRC can be expressed as:

(5) $$C_{S_{i},S_{j}}(\tau )=\frac{1}{L_{S}}\sum_{n=1}^{L_{S}}S_{i}\left(n\right)S_{j}\langle n+\tau \rangle =\left\{\begin{array}{cc} 0, & i\neq j\\ 1, & i=j,\;\tau =0\\ \lambda_{i}\left(\tau \right), & i=j,\;\tau =kL_{m}(k=1,\;\ldots ,\;L_{h})\\ \left(-1/L_{m}\right)\lambda_{i}\left(\tau \right), & i=j,\;\tau \neq kL_{m}(k=1,\;\ldots ,\;L_{h}) \end{array}\right.,$$

where we define <*n* + *τ*> as [(*n* + *τ*) mod *L_s_*], *λ_i_* (*τ*) is the auto-correlation value of ***H****_i_* that is used to generate ***S****_i_*, and |*λ_i_* (*τ*) ≤ 1|. For Equation (5), when *i* = *j*, *C**_S_**_i_*_, ***S****i*_ (*τ*) represents the periodic auto-correlation function (PACF) of ***S****_i_*. When *i* ≠ *j*, *C**_S_**_i_*_, ***S****j*_ (*τ*) represents the periodic cross-correlation function (PCCF) of ***S****_i_*. The OPRC has a zero-valued PCCF at any chip shift and a multi-valued PACF with high peaks equal to 1. Given the excellent correlation properties of OPRC, we further explore its potential in constructing ACDM-based VLP systems. We also develop a more advanced VLPC system design that integrates OPRC with the SICD scheme, which can leverage the unique characteristics of OPRC and SICD to mitigate MAI caused by asynchronous transmissions. The specific principles of the proposed system will be detailed in the following sections.

## 3. ACDM-Based VLP System by Using OPRC

### 3.1. System Principle of OPRC-VLP

**Figure 1:**
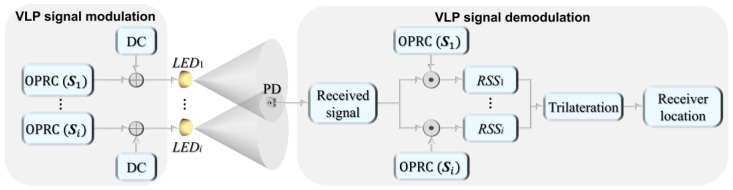
Schematic diagram of the ACDM-based VLP system using OPRC.

The schematic diagram of the ACDM-based VLP system is shown in Figure 1, where multiple LED transmitters are used to locate a PD receiver. Each LED transmitter is assigned a unique OPRC code ***S****_i_* of length *Ls*, serving as its AC signal. This signal is superimposed with a DC bias to generate the VLP signal for the *i*-th transmitter. Then, the LED transmitters continuously and periodically emit these VLP signals. As the VLP signal from the *i*-th LED reaches the receiver, it incurs a time delay *τ_i_*. Upon reception, we first capture a signal segment of length *L_S_* and then demodulate this segment using the OPRC that matches the transmitted one. This process accurately extracts the received signal strength (RSS) of the *i*-th LED transmitter. Once the RSS values of all LED transmitters are obtained, the trilateration algorithm is employed to determine the position of the receiver.

We define ***S****_i_* = [*S_i_*_1_, …, *S_LS_*], where *S_i_*_1_ ∈ [−1, +1], as the OPRC assigned to the *i*-th LED. Then, one period of the VLP signal from the *i*-th LED can be described as:

(6) $$T_{i}\left(t\right)=P_{t}\left(\alpha S_{i}\left(t\right)+1\right),\;0\leq t<nT_{c}L_{S},$$

where *P_t_* is the LED transmit power, *α* is the modulation index, and *T_c_* is the duration of one code chip. The VLP signals from different LEDs reach the receiver after experiencing different delays. One period of the received signal can be represented as:

(7) $$R\left(t\right)=\sum_{i=1}^{N_{\mathrm{LED}}}\beta \delta_{i}T_{i}\langle t+\tau_{i}\rangle +N_{\mathrm{AWGN}}\left(t\right),$$

where *N*_LED_ is the number of LEDs, *β* is the PD responsivity, *δ_i_* is the channel DC gain of the *i*-th VLP signal, and *N*_AWGN_ is additive white Gaussian noise (AWGN), whose variance is given by [35]:

(8) $$\sigma^{2}=2q\left(\sum_{i=1}^{N_{\mathrm{LED}}}\beta P_{t}\delta_{i}+I_{bg}I_{2}\right)B+8\pi kT_{k}\eta A_{r}B_{2}\left(\frac{I_{2}}{G}+\frac{2\pi \Gamma }{g_{m}}\eta A_{r}I_{3}B\right).$$

Here, *q* is the electronic charge, *I_bg_* is the background current, *I*_2_ is the noise bandwidth factor, *B* is the equivalent noise bandwidth (equal to system bandwidth), *k* is the Boltzmann constant, *T_k_* is the absolute temperature, *η* is the fixed capacitance of the PD per unit area, *G* is the open-loop voltage gain, *Γ* is the FET channel noise factor, *I*_3_ is the gate-induced drain leakage, and *g_m_* is the FET transconductance.

To simplify the system, we consider only the line-of-sight (LOS) component of the visible light channel, assuming that the LEDs face downward and the PDs face upward. Based on the Lambertian model in [35], the channel DC gain in Equation (7) can be expressed as:

(9) $$\delta_{i}=\frac{\left(m_{l}+1\right)A_{r}h^{m_{l}+1}g_{f}g_{c}}{2\pi D_{i}^{m_{l}+3}},$$

where *m_l_* is the Lambertian order, *A_r_* is the effective receiving area of PD, *h* is the vertical distance between each LED and the PD, *g_f_* is the gain of an optical filter, *g_c_* is the gain of an optical concentrator, and *D_i_* is the signal transmission distance from the *i*-th LED transmitter to the PD receiver.

Next, we sequentially perform cross-correlation operations between the received signal and the OPRC ***S****_i_* (*i* = 1, …, *N*_LED_) with different chip offsets *τ_k_* = (1, …, *L_S_*) to obtain their respective correlation values, given by:

(10) $$\begin{array}{l} CV_{ik}=\frac{1}{L_{S}}\sum_{n=1}^{L_{S}}R\left(n\right)S_{i}\langle n+\tau_{k}\rangle \\ =\frac{1}{L_{S}}\beta \sum_{j=1}^{N_{LED}}\sum_{n=1}^{L_{S}}\delta_{j}T_{j}\langle n+\tau_{j}\rangle S_{i}\langle n+\tau_{k}\rangle +\frac{1}{L_{S}}\sum_{n=1}^{L_{S}}S_{i}\langle n+\tau_{k}\rangle N_{\mathrm{AWGN}}\left(n\right)\\ =\alpha \beta P_{t}\sum_{j=1}^{N_{LED}}\left[\delta_{j}C_{S_{j},S_{i}}(\left|\tau_{j}-\tau_{k}\right|)+\sum_{n=1}^{L_{S}}\delta_{j}S_{i}\langle n+\tau_{k}\rangle \right]+\frac{1}{L_{S}}\sum_{n=1}^{L_{S}}S_{i}\langle n+\tau_{k}\rangle N_{\mathrm{AWGN}}\\ =\alpha \beta P_{t}\delta_{i}C_{S_{i},S_{i}}(\left|\tau_{i}-\tau_{k}\right|)+N_{\mathrm{MAI}}+{\tilde{N}}_{\mathrm{AWGN}},\\ \mathrm{where}\;N_{\mathrm{MAI}}=\alpha \beta P_{t}\sum_{j=1,\;j\neq i}^{N_{LED}}\delta_{j}C_{S_{j},S_{i}}(\left|\tau_{j}-\tau_{k}\right|),\;\\ \mathrm{and}\;{\tilde{N}}_{\mathrm{AWGN}}=\frac{1}{L_{S}}\sum_{n=1}^{L_{S}}S_{i}\langle n+\tau_{k}\rangle N_{\mathrm{AWGN}}. \end{array}$$

Here, $N_{\mathrm{MAI}}$ represents MAI caused by the interference of different OPRCs, and ${\tilde{N}}_{\mathrm{AWGN}}$ represents AWGN reduced by processing gain of OPRC. According to Equation (5), the correlation functions between different OPRCs are zero; thus, Equation (10) can be written by:

(11) $$CV_{ik}=\alpha \beta P_{t}\delta_{i}C_{S_{i},S_{i}}(\left|\tau_{i}-\tau_{k}\right|)+{\tilde{N}}_{\mathrm{AWGN}},$$

where ${\tilde{N}}_{\mathrm{AWGN}}$ represents the AWGN reduced by the coding gain of OPRC. Then, the RSS of the *i*-th LED is equal to the maximum of *CV_ik_*, written as:

(12) $$RSS_{i}=\max(CV_{ik})=\alpha \beta P_{t}\delta_{i}+{\tilde{N}}_{\mathrm{AWGN}}.$$

By substituting Equation (9) into the above equation and performing a straightforward transformation, we can derive the signal transmission distance from the *i*-th LED transmitter to the PD receiver from:

(13) $$D_{i}={\left[\frac{\left(m_{l}+1\right)A_{r}h^{m_{l}+1}g_{f}g_{c}\alpha \beta P_{t}}{2\pi \left(RSS_{i}-{\tilde{N}}_{\mathrm{AWGN}}\right)}\right]}^{1/\left(m_{l}+3\right)}.$$

Finally, after obtaining the signal transmission distance from each LED transmitter to the PD receiver, we can apply the trilateration algorithm to determine the receiver’s position. This process allows us to achieve VLP without MAI in the asynchronous system.

### 3.2. System Setup

**Figure 2:**
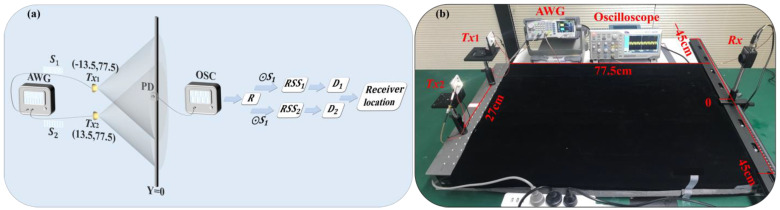
The ACDM-VLP system: (**a**) simulation model; and (**b**) experimental platform.

To validate the effectiveness of the proposed OPRC-based VLP scheme, we construct a simulation model and set up a corresponding experimental platform, as illustrated in Figure 2. The evaluation is conducted on a two-dimensional plane with dimensions of 90 cm × 77.5 cm, focusing on one-dimensional positioning. Table 1 lists the key parameters used during the simulation, which are closely approximated to those of the practical experimentation. Two LED transmitters, *Tx*_1_ at (−13.5 cm, 77.5 cm) and *Tx*_2_ at (13.5 cm, 77.5 cm), are used to determine the position of a PD receiver (*Rx*). The PD is placed on the *Y*-axis (Y = 0) and is moved along the *X*-axis from −44.5 cm to 44.5 cm in 5 cm intervals. Throughout this movement, the receiving plane of the PD remains parallel to the emitting planes of the LEDs.

For VLP signal modulation, we assign two unique OPRCs, labeled ***S***_1_ and ***S***_2_, each with a length of 128, to transmitters *Tx*_1_ and *Tx*_2_. We choose on–off keying (OOK) to modulate these codewords due to its simplicity and compatibility with ACDM. During simulation, these codewords serve as VLP signals from their respective transmitters. A DC bias is applied, and random time delays are introduced to simulate asynchronous transmission. For practical experimentation, a DG1062Z (RIGOL, Suzhou, China) arbitrary waveform generator (AWG) modulates ***S***_1_ and ***S***_2_ using OOK at 500 kHz with a 1 V peak-to-peak voltage. The modulated signals are then superimposed with a 3.2 V DC bias and fed into two Osram LCW W5SM LED light sources. Asynchronous transmission of VLP signals is achieved by successively activating the LED transmitters.

The asynchronous VLP signals travel through the VLC channel to reach the PD receiver, a PDA100A2 (THORLABS, Shenzhen, China) photodiode. Upon reception, the light signals undergo photoelectric conversion. The resulting electrical signals are sampled by a TBS 1202B (Tektronix, Shenzhen, China) oscilloscope (OSC) and then transferred to a computer for demodulation and analysis.

During the VLP demodulation phase, we sequentially perform cross-correlation calculations between the received VLP signals and ***S***_1_ and ***S***_2_ with various chip offsets. Using Equations (12) and (13), we determine the RSS and the signal transmission distance for each LED. By combining these measurements with the LED coordinate information, we apply the trilateration algorithm to locate the receiver.

### 3.3. Simulation and Experimental Results

**Figure 3:**
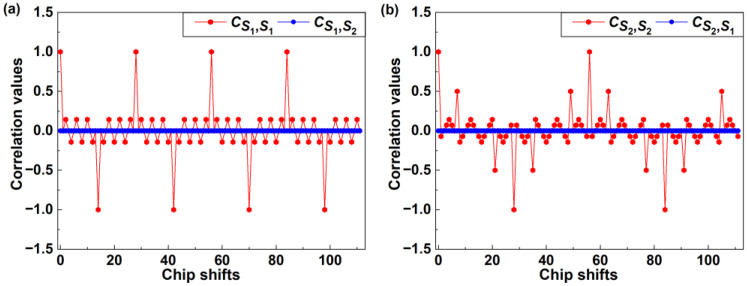
Correlation values at different chip shifts for (**a**) ***S***_1_; and (**b**) ***S***_2_.

**Figure 4:**
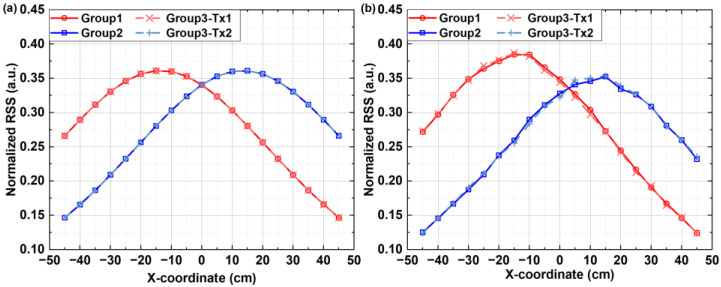
Estimated RSS of the ACDM-VLP system from: (**a**) simulation; and (**b**) experimental tests.

We begin by assessing the PCCF and PACF values of the OPRC codes ***S***_1_ and ***S***_2_ under different code chip offsets, as illustrated in Figure 3. It can be observed that the employed OPRC exhibits zero-valued PCCF and multiple-valued PACF characteristics, which are crucial for maintaining the orthogonality of VLP signals during asynchronous transmission. Next, we execute a series of simulations and experiments to test the VLP performance based on OPRC using ***S***_1_ and ***S***_2_. The tests are categorized into three groups. Specifically, in Groups 1 and 2, we activate transmitters *Tx*_1_ and *Tx*_2_ separately to analyze the received signal characteristics from individual sources. For Group 3, we successively activate *Tx*_1_ and *Tx*_2_ to evaluate the performance of the OPRC-VLP scheme under asynchronous transmission. The RSS measurements for *Tx*_1_ and *Tx*_2_ at various locations are recorded, and the outcomes are depicted in Figure 4. Here, the “Group3-*Tx_1_*” and “Group3-*Tx_2_*” curves represent the RSS fluctuations for each transmitter at different measurement points during the Group 3 test. From Figure 4, we observe discrepancies between the normalized RSS obtained from simulations and experiments. Both setups use LED lamps with power parameters set at 3 W. However, in the actual experiment, the power of *Tx*_1_ slightly exceeds 3 W due to variations in LED quality, while the power of *Tx*_2_ is slightly below 3 W. This leads to slightly higher normalized RSS measurements for *Tx*_1_ and slightly lower ones for *Tx*_2_ compared to the simulation results. Additionally, the ideal Lambertian model does not perfectly match the real radiation of the LEDs, and human measurement errors during the experiment may have exacerbated the numerical differences between simulation and experimental results. Despite these discrepancies, the overall trends remain consistent: both the simulated and measured normalized RSS values peak near ±13.5 cm, close to the LED positions, and then gradually decrease on either side. Notably, our comparative analysis demonstrates that, despite asynchronous signal transmission in the Group 3 test, we can accurately extract the RSS of *Tx*_1_ and *Tx*_2_ from the composite VLP signal in both simulation and experiment. The measurements closely match the single-source tests in Groups 1 and 2, thus strongly supporting the capability of the OPRC-VLP strategy to efficiently differentiate and precisely extract the VLP signals of individual LED transmitters during signal superposition and asynchronous transmission.

**Figure 5:**
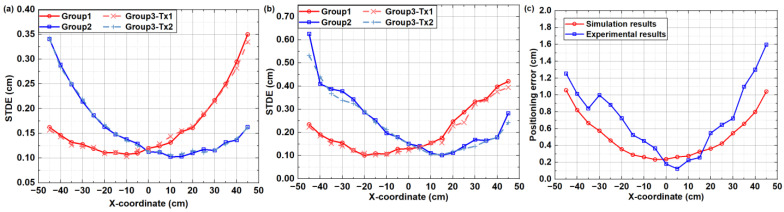
Distribution of VLP accuracy based on OPRC: (**a**) STDE in simulation; (**b**) STDE in experimental tests; and (**c**) positioning error.

After determining the RSS of each LED, we further calculate the corresponding signal transmission distances using Equation (13). However, due to inherent noise, the measured distances inevitably diverge from theoretical expectations. To quantify this discrepancy, we introduce the signal transmission distance error (STDE), defined as the absolute value of the difference between measured and theoretical distances. Figure 5a,b illustrates the STDE distributions across various measurement points for the three test groups. Owing to the perfect correlation properties of the OPRC, the STED measurements for *Tx*_1_ and *Tx*_2_ in Group 3 align closely with the individual test results from Group 1 and Group 2. This consistency confirms the results observed in the RSS patterns. Importantly, in areas close to the LEDs where the RSS is higher, the noise effect diminishes, leading to reduced STDE values. Additionally, given *Tx*_1_’s higher luminous intensity compared to *Tx*_2_, the STDE for *Tx*_1_ is generally smaller. This observation further confirms the negative correlation between signal strength and measurement error.

Based on the estimated signal transmission distances, Figure 5c illustrates the distribution of positioning errors. In the simulation environment, the system shows impressive positioning accuracy, with a maximum PE of approximately 1.10 cm and an average error of just 0.51 cm. In contrast, the experimental environment shows a slight decline in performance, with the maximum PE increasing to around 1.60 cm and the average PE rising to 0.72 cm. Notably, in both simulation and experimental tests, the highest positioning accuracy is observed near the origin of the *X*-axis (X = 0). This is attributed to the maximum received light intensity in this area, which minimizes the impact of noise on positioning accuracy. However, in the experimental tests, the weaker light output from *Tx*_2_ compared to *Tx*_1_ results in more significant positioning errors in the positive *X*-axis direction (away from *Tx*_2_) compared to the negative *X*-axis direction. Additionally, environmental noise and measurement errors lead to fluctuations in positioning accuracy during the experiment. For instance, the positioning error at the −30 cm position is approximately 0.2 cm greater than at the −35 cm position. Despite these challenges, our positioning system demonstrates strong robustness, with an average PE less than 1 cm. This confirms the effectiveness and accuracy of our proposed method, especially in asynchronous systems.

## 4. VLPC Network Based on SICD-OPRC Scheme

### 4.1. System Principle of SICD-OPRC

For the ACDM-OPRC-based VLP system design described in Section 3, OPRC sequences are cyclically broadcasted by LED transmitters. If we substitute plain cyclic OPRC sequences with OPRC-encoded user data at the transmission stage, the cross-correlation outcomes between received signals and OPRC sequences at the receiver can be utilized to encapsulate both RSS information and user data. This dual functionality allows us to extract the necessary RSS information from the cross-correlation results for VLP while also decoding user data for VLC by setting a reasonable threshold to parse the cross-correlation values. This method leverages the OPRC encoding mechanism to integrate VLP and VLC functions into a single system, constructing an integrated VLPC system.

**Figure 6:**
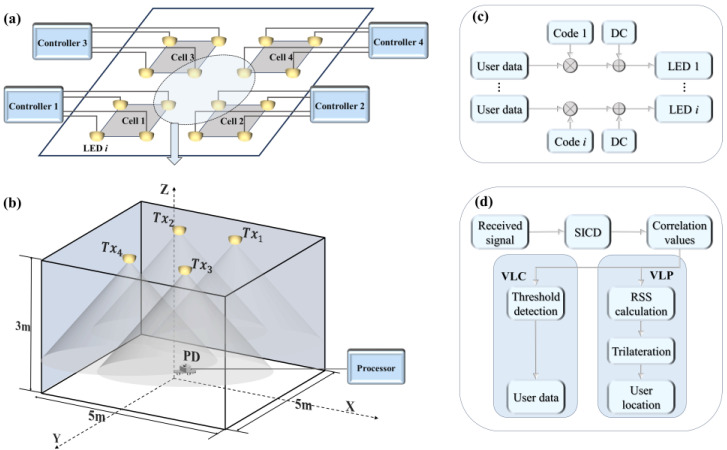
Schematic diagram of the VLPC network based on SICD-OPRC: (**a**) distribution of LED transmitters; (**b**) VLPC system model in the 4-LED cell; (**c**) VLPC signal modulation in the controllers at the LED transmitter side; and (**d**) VLPC signal demodulation in the processor at the PD receiver side.

Reflecting on the practical deployment of LEDs, we consider a multi-cell VLPC network architecture, as illustrated in Figure 6, with LEDs spaced equidistantly. Within this framework, LEDs in each cell are governed by a dedicated controller, ensuring synchronized signal transmission within the cell. In contrast, LEDs across different cells are managed by their respective controllers, leading to asynchronous signal transmission among them. Based on this architecture, user data are encoded by OPRCs, combined with a DC component to generate the VLPC signals, which are then distributed to every LED transmitter via the controller for broadcast. These VLPC signals traverse the VLC channel and are eventually captured by the PD receiver. The received signal can be expressed as:

(14) $$R_{s}\left(t\right)=\sum_{i=1}^{N_{\mathrm{LED}}}\beta \delta_{i}P_{t}\left[\alpha B_{i}\left(t+\tau_{i}\right)S_{i}\langle t+\tau_{i}\rangle +1\right]+N_{\mathrm{AWGN}}\left(t\right),$$

where ***B****_i_* and *τ_i_* represent the user data and time delay of the *i*-th VLPC signal emitted from the *i*-th LED, respectively. When the PD receives signals from synchronized LED transmitters, *τ_i_* = 0 for *i* = 1, …, *N*_LED_.

**Figure 7:**
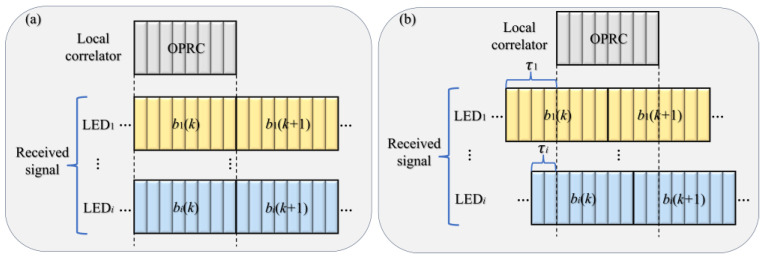
Schematic diagram of the decoding process for (**a**) synchronous transmission scenario; and (**b**) asynchronous transmission scenario.

Then, we use the OPRC corresponding to the *i*-th LED to decode the received signal, as shown in Figure 7. For the synchronous transmission scenario, the cross-correlation results between the received signal and OPRC for the *i*-th LED can be simply expressed as:

(15) $$\begin{array}{ll} CV_{Si}\left(k\right) & =\alpha \beta P_{t}\frac{1}{L_{S}}\sum_{j=1}^{N_{\mathrm{LED}}}\sum_{n=1}^{L_{S}}\delta_{j}b_{j}\left(k\right)S_{j}\left(n\right)S_{i}\left(n\right)+{\tilde{N}}_{\mathrm{AWGN}}\\ & =\alpha \beta P_{t}\delta_{i}\frac{1}{L_{S}}b_{i}\left(k\right)\sum_{n=1}^{L_{S}}S_{i}\left(n\right)S_{i}\left(n\right)+{\tilde{N}}_{\mathrm{AWGN}} \end{array},$$

where *b_j_*(*k*) is the *k*-th symbol of the user data from the *j*-th LED. As for the asynchronous transmission scenario, the cross-correlation results exhibit a more complex form, which can be expressed as:

(16) $$CV_{ASi}\left(k\right)=\alpha \beta P_{t}\frac{1}{L_{S}}\sum_{j=1}^{N_{\mathrm{LED}}}\delta_{j}\left[\begin{array}{l} \sum_{n=1}^{L_{S}-\tau_{j}}b_{j}\left(k\right)S_{j}\langle n+\tau_{j}\rangle S_{i}\left(n\right)+\\ \sum_{n=L_{S}-\tau_{j}+1}^{L_{S}}b_{j}\left(k+1\right)S_{j}\langle n+\tau_{j}\rangle S_{i}\left(n\right) \end{array}\right]+{\tilde{N}}_{\mathrm{AWGN}}.$$

For ease of expression, we assume that when recovering the signal transmitted by the *i*-th LED, the system can always accurately identify the start symbol of the signal. When adjacent bits of user data are the same, for example *b_i_*(*k*) = *b_i_*(*k* + 1) = +1, Equation (16) can be rewritten as follows:

(17) $$\begin{array}{ll} CV_{ASi}\left(k\right) & =\alpha \beta P_{t}\delta_{i}\frac{1}{L_{S}}\sum_{n=1}^{L_{S}}S_{i}\left(n\right)S_{i}\left(n\right)+\alpha \beta P_{t}\frac{1}{L_{S}}\sum_{\begin{array}{l} j=1,\\ j\neq i \end{array}}^{N_{\mathrm{LED}}}\delta_{j}\sum_{n=1}^{L_{S}}S_{j}\langle n+\tau_{j}\rangle S_{i}\left(n\right)+{\tilde{N}}_{\mathrm{AWGN}}\\ & =\alpha \beta P_{t}\delta_{i}\frac{1}{L_{S}}\sum_{n=1}^{L_{S}}S_{i}\left(n\right)S_{i}\left(n\right)+{\tilde{N}}_{\mathrm{AWGN}} \end{array}.$$

When adjacent bits of user data are different, for example *b_i_*(*k*) = +1 and *b_i_*(*k* + 1) = −1, then Equation (16) can be rewritten as follows:

(18) $$\begin{array}{ll} CV_{ASi}\left(k\right) & =\alpha \beta P_{t}\delta_{i}\frac{1}{L_{S}}\sum_{n=1}^{L_{S}}S_{i}\left(n\right)S_{i}\left(n\right)+{\tilde{N}}_{\mathrm{AWGN}}\\ & +\alpha \beta P_{t}\frac{1}{L_{S}}\sum_{\begin{array}{l} j=1,\\ j\neq i \end{array}}^{N_{\mathrm{LED}}}\delta_{j}\left[\sum_{n=1}^{L_{S}-\tau_{j}}S_{j}\langle n+\tau_{j}\rangle S_{i}\left(n\right)-\sum_{n=L_{S}-\tau_{j}+1}^{L_{S}}S_{j}\langle n+\tau_{j}\rangle S_{i}\left(n\right)\right] \end{array}.$$

In Equation (18), the third term represents the MAI caused by asynchronous transmission and the differences between adjacent bits of user data. We observe that not only does the chip offset weaken the orthogonal properties of VLPC signals, but the differences between user data bits further interfere with this orthogonality, thereby reducing the decoding accuracy. Thus, it is impossible to rely solely on the correlation properties of OPRC to avoid interference between different VLPC signals.

To address the challenges of asynchronous transmission and interference between adjacent user data bits in ACDM-based VLPC systems, we introduce and modify the multi-user interference cancellation method from [30] and apply it to mitigate the interference among signals from different LED transmitters, naming this scheme as successive interference cancellation decoding (SICD). This method aims to progressively eliminate interference signals to recover the user data and RSS corresponding to each VLPC signal accurately. The detailed implementation of the scheme is listed as follows.

- Step 1: Initial decoding and signal ordering

First, we use the OPRC corresponding to each VLPC signal to perform an initial decoding of the received signal to estimate the initial RSS of each VLPC signal. Next, we sort the VLPC signals in descending order based on their initial RSS values and designate the VLPC signal with the *m*-th largest RSS as the *m*-th VLPC signal.

- Step 2: Decoding the VLPC signal with the maximum RSS

We start by decoding the VLPC signal with the maximum RSS, while treating other VLPC signals as noise. We perform cross-correlation between the received signal and the corresponding OPRC. The obtained cross-correlation value can be expressed by:

(19) $$CV_{1}\left(k\right)=\frac{1}{L_{S}}\sum_{n=1}^{L_{S}}R_{s}\left(L_{S}\left(k-1\right)+{\overset{⌢}{\tau }}_{1}+n\right)S_{i}\left(n\right),$$

where ${\overset{⌢}{\tau }}_{i}$ represents the estimated time delay of the *i*-th VLPC signal, which can be obtained by adding a pilot to the beginning of the VLPC signal. Then, we set a threshold, setting as 0 in this paper, to judge the cross-correlation value and recover the user data carried by the 1-th VLPC signal. The process of recovering the *k*-th symbol of user data can be represented as:

(20) $$U_{1}\left(k\right)=\left\{\begin{array}{c} 1,\\ 0, \end{array}\;\begin{array}{c} \mathrm{if}\;CV_{1}\left(k\right)>0\\ \mathrm{if}\;CV_{1}\left(k\right)<0 \end{array}\right..$$

At the same time, the RSS of the 1-th VLPC signal is updated from:

(21) $$RSS_{1}=\left|CV_{1}\left(k\right)\right|.$$

- Step 3: Interference cancellation and subsequent signal decoding

We remove the decoded VLPC signal from the received signal. Then, the updated received signal after removing the (*m*−1)-th VLPC signal is:

(22) $${\overset{⌢}{R}}_{sm}={\overset{⌢}{R}}_{s\left(m-1\right)}-RSS_{m-1}U_{m-1}S_{m-1}.$$

Thus, the updated cross-correlation value of the *m*-th VLPC signal can be calculated from:

(23) $$CV_{m}\left(k\right)=\frac{1}{L_{S}}\sum_{n=1}^{L_{S}}{\overset{⌢}{R}}_{sm}\left(L_{S}\left(k-1\right)+{\overset{⌢}{\tau }}_{m}+n\right)S_{m}\left(n\right).$$

Then, we can use Equations (20) and (21) to recover the user data and RSS of the *m*-th VLPC signal. Therefore, by iteratively removing the decoded signals and updating the received signal, the SICD scheme gradually eliminates interference, allowing for the accurate decoding of each subsequent VLPC signal. This successive interference cancellation approach ensures that the decoding accuracy is maintained even in the presence of asynchronous transmissions and user data bit interference, thereby enhancing the VLC and VLP performance of the VLPC system.

### 4.2. Simulation Setup

To assess the coverage performance of the proposed SICD-OPRC-based VLPC scheme over a wide range of positions, we extend the model to a 5 m × 5 m × 3 m space, as shown in Figure 6b, which is different from our OPRC-VLP system. Due to the limited experimental space and equipment, we only conduct the simulation to validate the effectiveness of our proposed scheme. In the simulation model, four LED light sources are evenly positioned on the ceiling: *Tx*_1_ at (−1.25 m, 1.25 m, 3 m), *Tx*_2_ at (1.25 m, 1.25 m, 3 m), *Tx*_3_ at (−1.25 m, −1.25 m, 3 m), and *Tx*_4_ at (1.25 m, −1.25 m, 3 m). Each LED utilizes a distinct OPRC code for encoding user data. Next, we place 2601 test points evenly on the receiving plane, with each point separated by 10 cm. The receiver is placed on these test points, with its plane remaining parallel to the LED’s plane, at a constant vertical distance of 2.15 m. During simulations, the LED transmission power is set at 10 W, the system bandwidth is 1 GHz, and the decision threshold of the SICD algorithm is set at zero. Other parameters follow the settings in Table 1. We conduct a comprehensive evaluation of the performance of the SICD-OPRC-based VLPC scheme under both synchronous and asynchronous transmission environments. In synchronous mode, a single controller coordinates all LEDs for synchronized signal transmission. In contrast, asynchronous mode features each LED controlled independently, leading to varied signal transmission delays. The OPRC code and the OZCZ code from [26] are also adopted as benchmarks, and we compare them to evaluate how different coding schemes affect overall performance. These schemes are labeled as (i) SICD-OPRC, (ii) OPRC, and (iii) OZCZ.

We monitor and record the key performance parameters of the VLPC system under various conditions. First, we focus on assessing the efficacy of OPRC and OZCZ codes with varying lengths in mitigating MAI under asynchronous transmission conditions. This assessment is based on the computation of the normalized MAI generated by interfering codewords at different chip offsets relative to the target codeword. The formula for calculating this normalized MAI is given by:

(24) $$\xi \left(\tau \right)=\frac{1}{L_{S}}\sum_{\begin{array}{l} j=1,\\ j\neq i \end{array}}^{N_{\mathrm{LED}}}\left[\sum_{n=1}^{L_{S}-\tau }b_{+}S_{j}\langle n+\tau \rangle S_{i}\left(n\right)+\sum_{n=L_{S}-\tau +1}^{L_{S}}b_{-}S_{j}\langle n+\tau \rangle S_{i}\left(n\right)\right],$$

where *b*_+_ and *b*_−_ represent the “+1” and “−1” symbols, respectively, while ***S****_i_* and ***S****_j_* represent the target codeword and interfering codeword, respectively. This equation quantifies the interference caused by the interfering codeword at different offsets, enabling us to evaluate the MAI suppression capabilities of the OPRC and OZCZ codes. Based on Equations (20) and (21), the performance of VLC and VLP is directly related to the accuracy of the measured CV values, i.e., the cross-correlation values *CV_m_*(*k*). Therefore, we further investigate the effectiveness of different coding schemes in combating MAI by evaluating the measurement error of the CV values. This is quantified by calculating the normalized error of CV (NECV) using the following formula:

(25) $$\zeta =\sum_{i=1}^{N_{\mathrm{LED}}}\frac{E\left(CV_{i}\right)}{\left|CV_{i}\right|},$$

where *E*(*CV_i_*) is the absolute difference between the measured and ideal CV values for the *i*-th VLPC signal. This deviation is caused by MAI and AWGN. Finally, to achieve a thorough assessment, we conduct performance evaluations considering specific metrics including BER and PE.

### 4.3. Simulation Results

**Figure 8:**
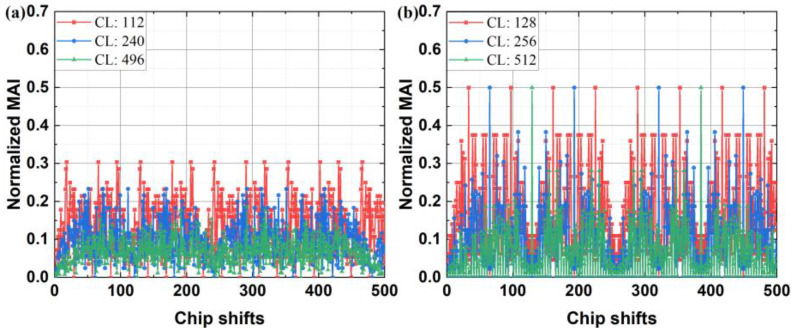
Normalized MAI in the asynchronous VLPC system using: (**a**) OPRC; and (**b**) OZCZ, with varying codeword lengths.

First, in Figure 8, we compare the normalized MAI under asynchronous transmission conditions for the OPRC and OZCZ schemes, considering varying codeword lengths (CLs). Here, we assume that the adjacent bit information in user data varies. The results indicate that normalized MAI decreases with increasing chip length, suggesting that longer codes are more effective in reducing MAI. Additionally, OPRC demonstrates a significantly lower MAI compared to OZCZ, highlighting its superior MAI suppression capability. Despite these benefits, residual interference remains a challenge for CV measurements. To address this, we introduce the SICD scheme to further mitigate the adverse effects of MAI on CV accuracy.

**Figure 9:**
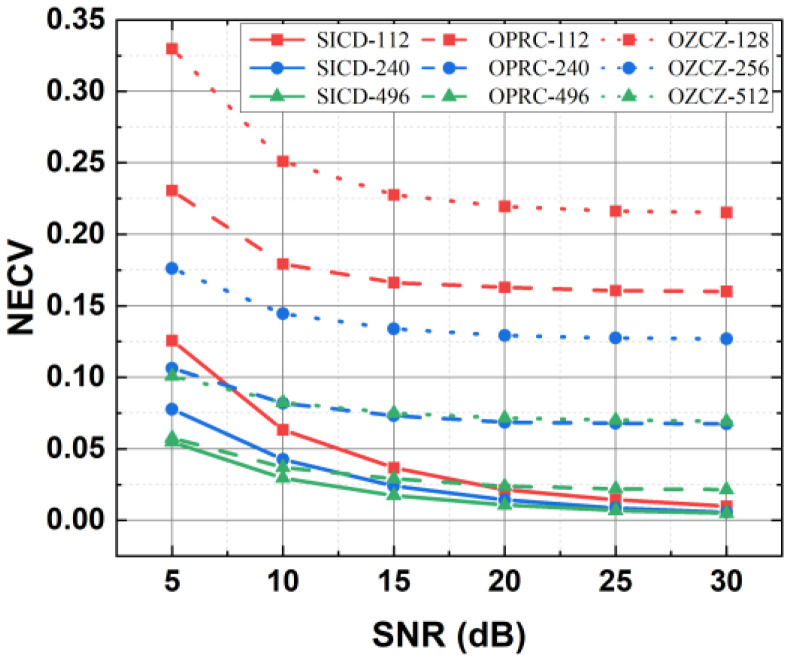
Estimated NECV by using different coding mechanisms with varying codeword lengths and SNRs when the receiver is located at (1.25 m, 1.25 m, 0 m).

Then, to verify the ability of SICD-OPRC to mitigate MAI on CV measurement and its robustness against AWGN, we fix the receiver at (1.25 m, 1.25 m, 0 m) and measure the NECV values for three coding strategies under varying degrees of AWGN, represented by different SNR levels. Figure 9 illustrates how the NECV values change with SNR for different coding strategies and codeword lengths. Across all test conditions, the SICD-OPRC strategy consistently exhibits the lowest NECV, demonstrating its effectiveness in mitigating the MAI in complex asynchronous transmission environments and achieving more precise CV measurements. Additionally, OPRC shows notably lower NECV values compared to OZCZ, reinforcing its superior MAI suppression capacity, as previously observed. In addition, the NECV values for all strategies increase with decreasing SNR, indicating that higher AWGN levels affect CV estimation. However, we find that longer codeword lengths typically result in lower NECV, especially in low SNR environments. This is because longer codewords are more effective at suppressing MAI and have a higher coding gain to mitigate the impact of AWGN [36]. For example, at an SNR of just 5 dB, the NECV value decreases from 0.126 for a 112-length OPRC to 0.078 for a 496-length OPRC. Therefore, in environments with poor SNR, using longer codeword lengths can improve system performance. However, increasing the codeword length also reduces the transmission rate, as more chip resources are required per bit. To balance optimal data throughput and MAI suppression, we select OPRC with a length of 112 and OZCZ with a length of 128 for subsequent system performance tests.

**Figure 10:**
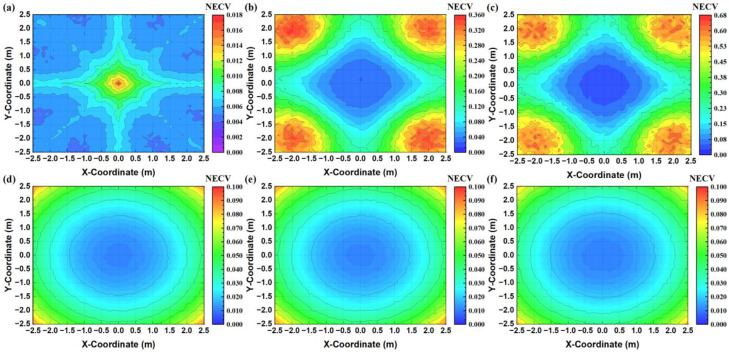
Color-mapped contour plots of NECV caused by MAI when using: (**a**) SICD-OPRC; (**b**) OPRC; and (**c**) OZCZ; color-mapped contour plots of NECV caused by AWGN when using: (**d**) SICD-OPRC; (**e**) OPRC; and (**f**) OZCZ.

Next, we position the receiver at various measurement points and calculate CV values according to Equation (18). Recognizing that both MAI and AWGN impact CV measurement, we quantify their individual contributions to measurement errors under differing coding mechanisms. The outcomes are depicted in color-mapped contour plots in Figure 10. Figure 10a–c clearly shows that across the entire testing area, the SICD-OPRC scheme results in substantially lower NECV due to MAI compared to the OPRC and OZCZ schemes. Interestingly, the NECV distribution pattern for SICD-OPRC differs from that of OPRC and OZCZ, with NECV values increasing as the receiver moves towards the center of the model, whereas OPRC and OZCZ exhibit a decreasing trend. This peculiarity arises because, at the center of the model, the signal strengths from the four LEDs are nearly uniform. This uniformity makes it challenging to identify the strongest VLPC signal during the SICD process, thereby reducing the scheme’s effectiveness in canceling interference. Thus, the performance of SICD is more effective at the peripheries of the model compared to the central area. Additionally, Figure 10d–f reveals a consistent NECV distribution pattern caused by AWGN: NECV values are lower near the center of the room and increase toward the edges, regardless of the coding scheme. This is because the received light intensity is higher in the center of the model, leading to a higher SNR, which mitigates the negative impact of AWGN. Notably, OZCZ exhibits slightly lower NECV values compared to the alternatives, likely due to the longer OZCZ codes providing a marginal coding gain that reduces AWGN effects. Overall, MAI-induced NECV is significantly less pronounced than the impact of AWGN. For OPRC and OZCZ schemes, MAI is the primary contributor to CV measurement errors, underscoring the effectiveness of the SICD-OPRC scheme in substantially reducing MAI effects.

**Figure 11:**
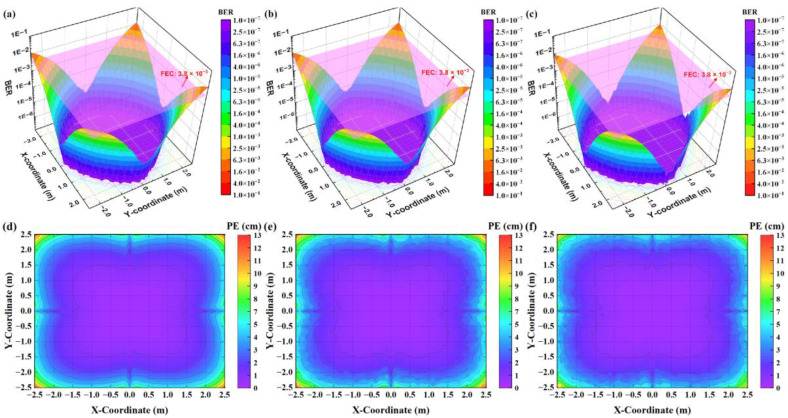
For the synchronous VLPC system, the color-mapped surface plots of BER estimated at all test points when using: (**a**) SICD-OPRC; (**b**) OPRC; and (**c**) OZCZ, with the pink plane representing the FEC threshold of 3.8 × 10^−3^, and the color-mapped contour plots of PE estimated at all test points when using: (**d**) SICD-OPRC; (**e**) OPRC; and (**f**) OZCZ.

**Figure 12:**
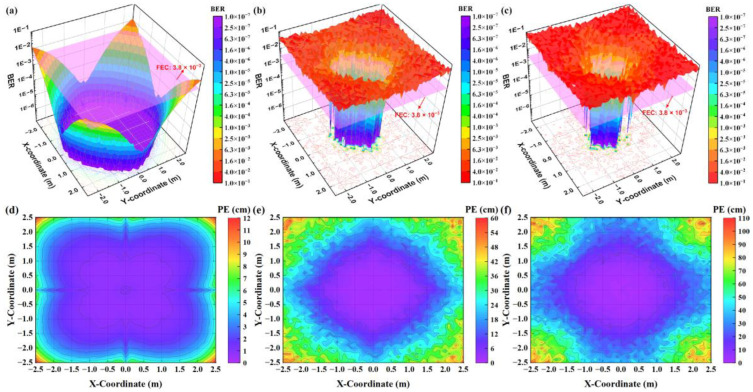
For the asynchronous VLPC system, the color-mapped surface plots of BER estimated at all test points when using (**a**) SICD-OPRC; (**b**) OPRC; and (**c**) OZCZ, with the pink plane representing the FEC threshold of 3.8 × 10^−3^, and the color-mapped contour plots of PE estimated at all test points when using: (**d**) SICD-OPRC; (**e**) OPRC; and (**f**) OZCZ.

Finally, we compare the VLPC system’s performance using the three coding schemes in both synchronous and asynchronous scenarios. We measure the BER and PE of the receiver at each test point and present the results in color-mapped surface plots and contour plots in Figure 11 and Figure 12. In the synchronous setting, Figure 11 illustrates that all three methods deliver similar VLC and VLP performance, achieving BER below the forward error correction (FEC) threshold of 3.8 × 10^−3^ and PE less than 10 cm across most test points. This similarity is due to the ability of these coding mechanisms to maintain signal orthogonality under synchronous conditions. Therefore, the decoding process is mainly affected by AWGN, resulting in a spatial distribution of BER and PE that mirrors the NECV distribution caused by AWGN shown in Figure 10d–f. That is, BER and PE are lower near the center of the model and higher at the edges. In asynchronous scenarios, Figure 12 demonstrates that the system employing the SICD-OPRC scheme sustains stable performance, meeting the FEC standard for VLC and achieving PE below 10 cm at most locations. By contrast, OPRC and OZCZ exhibit significant BER and PE increases, particularly at distant test points from the model’s center. The BER for OPRC can rise to 4.0 × 10^−2^ with PE nearing 60 cm, and OZCZ can reach a BER of 1.0 × 10^−1^ with PE approaching 110 cm. This difference is due to SICD-OPRC’s effectiveness in mitigating MAI, allowing it to accurately distinguish VLPC signals from different LEDs within complex received signals, thereby preserving performance despite asynchronous transmission-induced MAI. OPRC outperforms OZCZ due to its superior correlation characteristics, offering enhanced MAI resilience and superior VLPC results.

**Figure 13:**
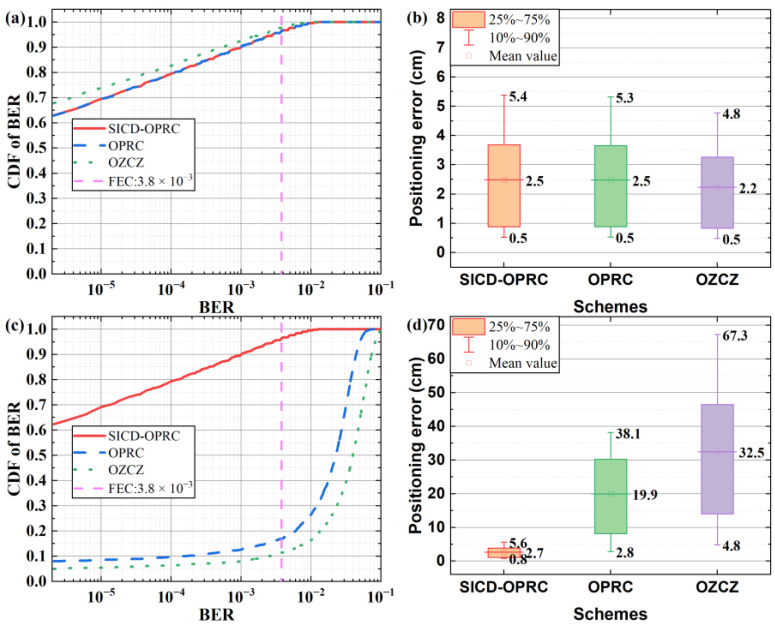
Statistical results of: (**a**) BER for the synchronous system; (**b**) PE for the synchronous system, (**c**) BER for the asynchronous system; and (**d**) PE for the asynchronous system.

For a quantitative comparison of each scheme’s performance, we conduct a statistical analysis of the BER and PE distributions depicted in Figure 11 and Figure 12. The summarized findings are presented in Figure 13. Under ideal synchronous transmission, all schemes perform well, achieving very similar performance levels. Specifically, about 97% of test points achieve a BER below the FEC threshold, while PE is effectively controlled below 5.4 cm for 90% of measurement positions. However, significant performance differences emerge when the transmission environment shifts to asynchronous operation. The system based on the OZCZ scheme exhibits a noticeable decline in performance, maintaining BER within the FEC standard at only 11% of test points, with average BER and PE reaching 4.0 × 10^−2^ and 32.5 cm, respectively. This is attributed to the OZCZ scheme’s limited resistance to MAI induced by asynchronous transmission. Compared to the OZCZ scheme, the OPRC scheme demonstrates relatively better performance, reducing the average BER and PE to 2.3 × 10^−2^ and 19.9 cm, with approximately 17% of test points complying with the FEC standard. Most strikingly, the SICD-OPRC scheme excels under asynchronous conditions. This scheme not only reduces average BER and PE to 4.3 × 10^−4^ and 2.7 cm but also achieves BER compliance with the FEC standard in approximately 96% of test points. Additionally, the PE does not exceed 5.6 cm for 90% of test points, nearly matching the performance of synchronous transmission systems. This result demonstrates the SICD-OPRC scheme’s effective suppression of MAI in asynchronous environments, precise separation of VLPC signals from different LEDs, and ensures high-performance VLC and VLP services even in the presence of MAI.

## 5. Conclusions

This paper investigated ACDM-based VLPC networks. We first proposed OPRC to enhance the orthogonality of VLP signals under asynchronous transmissions, thereby improving the reliability and efficiency of VLP systems. Through experiments, the OPRC-VLP scheme demonstrated its capability to achieve sub-centimeter positioning accuracy without requiring synchronization among transmitters. Furthermore, we extended the OPRC concept to simultaneously provide VLC and VLP services through the SICD-OPRC-VLPC scheme. This scheme combines the advantages of OPRC with SICD to eliminate MAI caused by asynchronous transmissions, enhancing data transmission quality and positioning accuracy in ACDM-based VLPC systems. Numerical results showed that the SICD-OPRC-VLPC system significantly reduces BER and PE compared to existing VLPC approaches like OZCZ, nearly matching the performance observed in synchronized systems. The system achieves an average BER of 4.3 × 10^−4^ and PE of 2.7 cm, with BER meeting the FEC standard at around 96% of test points and PE staying below 5.6 cm for 90% of test points. This study offers insights into VLPC for future 6G infrastructures, paving the way for enhanced multi-service capabilities such as lighting, communication, and positioning. Our future work will optimize the VLPC network architecture to accommodate various real-world conditions, including multipath reflections from the walls, receiver mobility across cell boundaries, and different LED layouts. In addition, the trade-off between data rate, system complexity, and positioning accuracy under higher-order modulation formats will be investigated.

## Figures and Tables

**Table 1:** Key parameters used in the simulation setup.

Parameters	Symbol	Values
Coordinates of LEDs	*Tx*_1_, *Tx*_2_	(−13.5 cm, 77.5 cm), (13.5 cm, 77.5 cm)
LED transmit power	*P_t_*	3 W
Modulation index	α	0.15
Number of the LED transmitter	N_LED_	2
Photodetector responsivity	*β*	0.4 A/W
System bandwidth	*B*	500 kHz
Physical area of *PD*	*A_r_*	75.4 mm^2^
Lambertian order of emission	m_l_	1
Gain of an optical filter	*g_f_*	1.5
Gain of an optical concentrator	*gc*	1

## Data Availability

Data underlying the results presented in this paper are not publicly available at this time but may be obtained from the authors upon reasonable request.
